# Efficacy and safety of bexagliflozin compared with dapagliflozin as an adjunct to metformin in Chinese patients with type 2 diabetes mellitus: A 24‐week, randomized, double‐blind, active‐controlled, phase 3 trial

**DOI:** 10.1111/1753-0407.13526

**Published:** 2024-04-07

**Authors:** Lingding Xie, Jie Han, Zhifeng Cheng, Dexue Liu, Jie Liu, Chunrong Xu, Wenli Sun, Qingju Li, Fang Bian, Wei Zhang, Jinyu Chen, Qian Zhu, Tara K. Thurber, J. Paul Lock, Bo Zhang

**Affiliations:** ^1^ China‐Japan Friendship Hospital Beijing China; ^2^ Hebei PetroChina Central Hospital Langfang China; ^3^ Fourth Hospital of Harbin Medical University Harbin China; ^4^ The First Affiliated Hospital of Nanyang Medical College Nanyang China; ^5^ Henan University of Science and Technology Affiliated First Hospital Luoyang China; ^6^ Xuzhou Cancer Hospital Xuzhou China; ^7^ Yueyang People's Hospital Yueyang China; ^8^ The Second Affiliated Hospital of Zhengzhou University Zhengzhou China; ^9^ Cangzhou People's Hospital Cangzhou China; ^10^ Newsoara Biopharma Co., Ltd Shanghai China; ^11^ TheracosBio, LLC Marlborough Massachusetts USA; ^12^ MetroWest Medical Center Natick Massachusetts USA

**Keywords:** bexagliflozin, comparison, dapagliflozin, noninferiority

## Abstract

**Background:**

Bexagliflozin and dapagliflozin are sodium‐glucose cotransporter‐2 (SGLT2) inhibitors. No direct comparison of SGLT2 inhibitors in a randomized controlled trial has been reported to date.

**Methods:**

This was a multicenter, randomized, double‐blind, active‐controlled trial comparing bexagliflozin to dapagliflozin for the treatment of type 2 diabetes mellitus in adults with disease inadequately controlled by metformin. Subjects (*n* = 406) were randomized to receive bexagliflozin (20 mg) or dapagliflozin (10 mg) plus metformin. The primary endpoint was noninferiority of bexagliflozin to dapagliflozin for the change in glycated hemoglobin (HbA1c) from baseline to week 24. Secondary endpoints included intergroup differences in fasting plasma glucose (FPG), 2‐h‐postprandial glucose (PPG), body weight, and systolic blood pressure (SBP) from baseline to week 24. The trial also evaluated the safety profiles.

**Results:**

The model‐adjusted mean change from baseline to week 24 HbA1c was −1.08% for bexagliflozin and −1.10% for dapagliflozin. The intergroup difference of 0.03% (95% confidence interval [CI] −0.14% to 0.19%) was below the prespecified margin of 0.4%, confirming the noninferiority of bexagliflozin. The changes from baseline in FPG, PPG, body weight, and SBP were −1.95 mmol/L, −3.24 mmol/L, −2.52 kg, and −6.4 mm Hg in the bexagliflozin arm and −1.87 mmol/L, −3.07 mmol/L, −2.22 kg, and −6.3 mm Hg in the dapagliflozin arm. Adverse events were experienced in 62.6% and 65.0% and serious adverse events affected 4.4% and 3.5% of subjects in the bexagliflozin and dapagliflozin arm, respectively.

**Conclusions:**

Bexagliflozin showed nearly identical effects and a similar safety profile to dapagliflozin when used in Chinese patients on metformin.

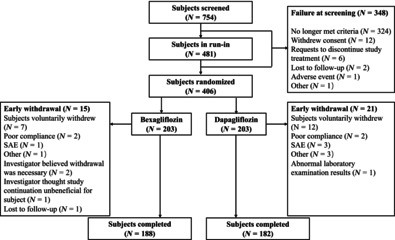

## Graphical abstract

Highlights

- This is the first randomized, double‐blind, controlled clinical trial collecting data on the efficacy and safety of bexagliflozin compared to that of dapagliflozin in Chinese patients with type 2 diabetes mellitus.
- Bexagliflozin provides identical efficacy in improving key efficacy parameters including glycated hemoglobin, fasting plasma glucose, 2‐h postprandial blood glucose, and clinical response without an increase in adverse events compared with dapagliflozin.

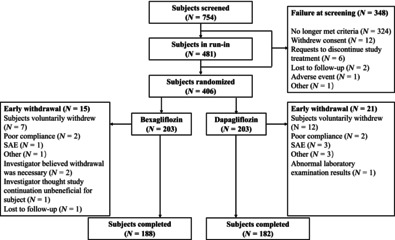

## 1 INTRODUCTION

Metformin is typically the first‐line therapy for managing type 2 diabetes mellitus (T2DM) due to its effective glucose‐lowering effects, favorable cost–benefit ratio, widespread availability, and extensive clinical history.^1^ As T2DM progresses, controlling the disease often requires adding one or more drugs as adjunctive therapy. Sodium‐glucose cotransporter‐2 (SGLT2) inhibitors are a promising class of oral hypoglycemic agents for T2DM patients with inadequate glycemic control. SGLT2 is a renal‐specific integral membrane protein responsible for reabsorption of most of the glucose passing through the S1 and S2 segments of the renal proximal tubule. SGLT2 inhibitors reduce plasma glucose in an insulin‐independent manner.^2^, ^3^ This mechanism of action makes SGLT2 inhibitors suitable for patients with varying degrees of beta‐cell function and insulin resistance. In addition to the hypoglycemic action, SGLT2 inhibitors have demonstrated favorable effects on body weight, blood pressure, and renal and cardiovascular function.^4^, ^5^ Large‐scale cardiovascular outcomes studies have shown that SGLT2 inhibitors can reduce cardiovascular events, particularly hospitalization for heart failure in patients with T2DM and heart failure with reduced ejection fraction, with or without diabetes.^6^, ^7^, ^8^, ^9^ Recently, SGLT2 inhibitors have also demonstrated beneficial effects in delaying the progression of kidney disease.^10^

Bexagliflozin is a potent selective inhibitor of human SGLT2 intended for the treatment of hyperglycemia in adults with T2DM.^11^, ^12^ The safety and effectiveness of bexagliflozin when deployed as a monotherapy or in combination with background hypoglycemic medications have been studied in subjects with T2DM with and without renal impairment.^13^, ^14^, ^15^, ^16^, ^17^, ^18^ In a phase 2b multicenter, double‐blind, placebo‐controlled, dose range finding study, the treatment effect of bexagliflozin as monotherapy, measured as placebo‐corrected least square means change in glycated hemoglobin (HbA1c) from baseline to week 12, was −0.55%, −0.68%, and −0.80% in the 5, 10, and 20 mg groups, respectively. The reduction in HbA1c was statistically significant (*p* < .0001) and clinically meaningful in all three bexagliflozin groups.^15^ In a phase 2, 96‐week multicenter, randomized, double‐blind, parallel‐group, monotherapy study, the mean change from baseline in HbA1c was −0.39% in the bexagliflozin arm and 0.49% in the placebo arm at week 24. The intergroup difference was significant, −0.79% (95% confidence interval [CI] −1.06 to −0.53; *p* < .0001). At week 96, the change in HbA1c was −0.55% in the bexagliflozin arm and 0.53% in the placebo indicating sustained improvement in HbA1c.^16^ In a phase 3, double‐blind, placebo‐controlled study to evaluate the efficacy and safety of bexagliflozin tablets, 20 mg in T2DM subjects inadequately controlled by metformin alone, the placebo‐corrected treatment effect was −0.53% (95% CI −0.74 to −0.32; *p* < .0001).^19^ In a phase 3 double‐blind, placebo‐controlled trial conducted in T2DM patients with moderate renal impairment (Stage 3 chronic kidney disease, 30 ≤ glomerular filtration rate < 60 mL/min per 1.73 m^2^), bexagliflozin reduced HbA1c, body weight, systolic blood pressure (SBP), and albuminuria.^18^ Patients in this trial continued their preexisting antidiabetic regimens with or without insulin throughout trial participation. Bexagliflozin was noninferior to sitagliptin^17^ or glimepiride^13^ as adjunct to metformin for reduction of HbA1c in phase 3 studies and was superior to both agents for reduction of fasting plasma glucose (FPG) and body weight, and superior to glimepiride for reduction of SBP and incidence of hypoglycemia.^13^

Dapagliflozin is widely used for the treatment of T2DM. The study was designed to determine whether the efficacy of bexagliflozin would be noninferior to that of the maximum approved dosage of dapagliflozin (10 mg) when used in combination with metformin hydrochloride. To the best of our knowledge, this Phase 3 study was the first randomized, double‐blind, head‐to‐head comparative investigation of efficacy and safety between two SGLT‐2 inhibitors in T2DM patients.

## 2 METHODS

### 2.1 Study design

The study was a 24‐week, multicenter, randomized, parallel, double‐blind, double‐dummy, active‐controlled phase 3 trial to compare the safety and efficacy of bexagliflozin, 20 mg to dapagliflozin, 10 mg in adults with T2DM inadequately controlled by metformin monotherapy. The study was conducted at 36 sites in China between March 2021 and December 2022. The study was registered as NCT05159882 on clinicaltrials.gov and complied with the ethical principles of the Declaration of Helsinki, the approved study protocol, Good Clinical Practice, and applicable regulations. The protocol and its revisions, as well as the written informed consent form for subjects, were approved in writing by a Clinical Research Ethics Committee prior to the start of the trial. Before any trial‐related procedures were carried out, all subjects signed an informed consent form.

### 2.2 Study population

Subjects with a history of T2DM, aged 18 or older, were eligible if they had been prescribed a daily dose of metformin ≥1500 mg that had not changed in the 8 weeks before screening. During the trial, the sponsor supplied metformin and subjects maintained consistent doses throughout the study period. They were required to have a % HbA1c of between 7.0% and 10.5% (inclusive), a body mass index (BMI) between 19 and 35 kg/m^2^ (inclusive), and unaltered treatment regimens for dyslipidemia or hypertension for 30 days prior to the screening visit.

Prospective subjects were ineligible for the following reasons: (a) a history of diabetes insipidus; (b) poorly controlled diabetes symptoms (such as significant polyuria and polydipsia); (c) a history of genital or urinary tract infection, including genital mycotic infection, within 6 weeks of screening or a history of ≥three genital or urinary tract infections requiring treatment within 6 months of screening; (d) hyperglycemia (fasting self‐monitored blood glucose values ≥13.9 mmol/L); (e) a history of diabetic ketoacidosis or hyperosmolar nonketotic coma; (f) severe osteoporotic fracture; (g) poorly controlled hypertension; (h) unstable weight due to surgery; (i) unstable endocrine/psychiatric/rheumatic disease; (j) risk of dehydration or fluid depletion; (k) alcohol/drug abuse within the past 6 months; (l) a diagnosis of type 1 diabetes, maturity‐onset diabetes of the young, or secondary diabetes; or (m) a history, within 6 months prior to screening, of any of the following: myocardial infarction, cardiac surgery or vascular reconstruction, unstable angina, unstable congestive heart failure, New York Heart Association congestive heart failure stage III or IV, transient ischemic attack or severe cerebrovascular disease, or unstable or poorly controlled arrhythmias. At screening, subjects could not have aspartate aminotransferase (AST) and/or alanine aminotransferase (ALT) ≥ threefold the upper limit of normal (ULN), total bilirubin ≥twofold the ULN, or estimated glomerular filtration rate <60 mL/min/1.73 m^2^. Table S1, containing the full exclusion criteria, is available as Supplementary Material.

### 2.3 Study medication and treatment

Subjects who met all the inclusion and exclusion criteria were enrolled in a 4‐week single blind run‐in period and instructed to consume two investigational products, bexagliflozin tablets, placebo and dapagliflozin tablets, placebo once a day in addition to their existing metformin regimen. After the run‐in period, eligible subjects were randomized to receive bexagliflozin tablets, 20 mg or bexagliflozin tablets, placebo, and dapagliflozin tablets, 10 mg or dapagliflozin tablets, placebo for 24 weeks of treatment. Subjects were monitored for efficacy and safety at weeks 4, 8, 12, 16, 20, and 24 during treatment, with a safety follow‐up visit at week 26 or upon early withdrawal from the study.

### 2.4 Statistical analysis

The sample size calculation was based on a two‐group *t* test with a one‐sided significance at the 2.5% level, assuming the noninferiority margin for the difference between from bexagliflozin group and the dapagliflozin group for the mean change from baseline to week 24 in HbA1c would be 0.4%, and the SD for the change in HbA1c from baseline to week 24 in each group would be 1.0%. Under these assumptions, an estimated sample size of 156 subjects per treatment group yielded ~90% power to establish noninferiority at the 2.5% significance level. A cohort size of ~195 subjects was considered adequate to account for potential subject attrition. Statistical analyses were performed using SAS® version 9.4.

### 2.5 Efficacy endpoints

The primary objective of the trial was to demonstrate that bexagliflozin was noninferior to dapagliflozin for the reduction in HbA1c from baseline to week 24. The null hypothesis held that the upper limit of the 95% CI for the difference in HbA1c between the treatment effects of bexagliflozin and dapagliflozin would be greater than or equal to 0.4%. The treatment effect analysis was based on a mixed‐effects model for repeated measures (MMRM) with multiple imputation. The dependent variable was the change in HbA1c from baseline, and the fixed‐effect covariates included treatment group, visit, the interaction between treatment group and visit, and baseline HbA1c value. The intention‐to‐treat (ITT) population (*n* = 406) was used for the primary endpoint analysis and a per‐protocol (PP) population (*n* = 349) was used for a sensitivity analysis.

The secondary efficacy analyses included descriptive statistics of the change from baseline to week 24 in FPG, 2‐h postprandial plasma glucose (PPG), body weight, and blood pressure. All observed efficacy data were analyzed using an MMRM analysis of covariance with the baseline HbA1c as the dependent variable, treatment group, visit, the interaction between treatment group and visit as independent variables, and the subject baseline HbA1c value of the dependent variable as a fixed‐effect covariate. Corresponding 95% CIs and *p* values were provided. All analyses were conducted in the ITT population.

### 2.6 Analysis of safety

The Medical Dictionary for Regulatory Activities version 25.1 was used to code adverse events (AEs), and the number (n) and percentage (%) of subjects who experienced adverse events were tabulated. AEs were categorized as: treatment‐emergent AEs (TEAEs), events related to drug exposure, serious AEs (SAEs), AEs of special interest (AESI), AEs that led to death, AEs that led to dosing cessation, and AEs that led to study withdrawal. Event severity was also recorded. Other safety analyses included evaluation of hypoglycemic episodes, vital signs, 12‐lead electrocardiograms, physical examinations, and results of laboratory testing. The safety analysis set consisted of the 406 subjects who received at least one dose of study medication following randomization.

## 3 RESULTS

### 3.1 Patient disposition and baseline

**FIGURE 1:**
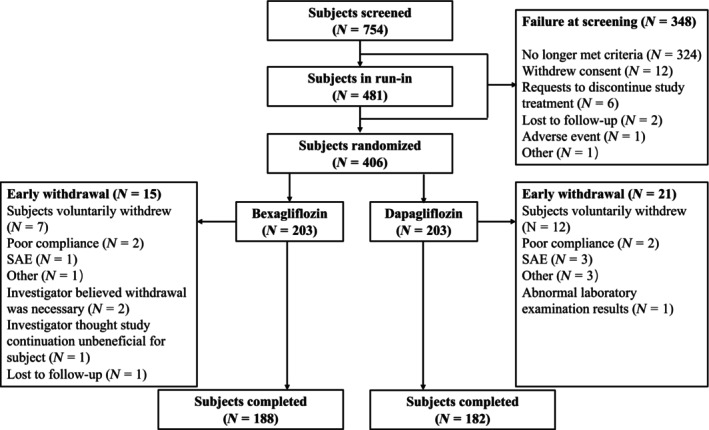
Disposition of subjects. SAE, serious adverse event.

Of the 754 subjects screened, 406 subjects were randomized to receive bexagliflozin, 20 mg or dapagliflozin, 10 mg, with 203 subjects assigned to each group. Of the randomized subjects, 370 (91.1%) completed the entire trial, including 188 subjects (92.6%) in the bexagliflozin group and 182 subjects (89.7%) in the dapagliflozin group. A schematic diagram of the subject allocation and disposition is presented in Figure 1.

The baseline characteristics for the ITT set are presented in Table 1. Subjects in the ITT analysis set were predominantly Chinese (99.3%) and Han ethnicity (97.3%). The ITT analysis set included 179 females (44.1%) and 227 males (55.9%). The mean age was 56.0 years, height was 163.9 cm, and weight was 70.2 kg. The mean BMI was 26.1 kg/m^2^. The demographic characteristics of the subjects were balanced in terms of age, race, ethnicity, height, and weight, but the proportion of female subjects was higher in the dapagliflozin group than in the bexagliflozin group, at 49.3% and 38.9%, respectively.

**TABLE 1:** Baseline characteristics.

Variables	Bexagliflozin (*N* = 203)	Dapagliflozin (*N* = 203)	Total (*N* = 406)
Age in years (SD)	56.2 (9.8)	55.9 (8.9)	56.0 (9.3)
Male (%)	124 (61.1)	103 (50.7)	227 (55.9)
Female (%)	79 (38.9)	100 (49.3)	179 (44.1)
Asian	203 (100)	200 (98.5)	403 (99.3)
Other	0	3 (1.5)	3 (0.7)
Disease duration in years (SD)	6.6 (5.3)	6.4 (4.8)	6.5 (5.1)
Diabetes complications, *n* (%)			
Yes	49 (24.1)	51 (25.1)	100 (24.6)
No	154 (75.9)	152 (74.9)	306 (75.4)
Body mass in kg (SD)	70.3 (11.9)	70.1 (11.6)	70.2 (11.7)
BMI in kg/m^2^ (SD)	26.0 (3.0)	26.1 (3.3)	26.1 (3.2)
HbA1c in % (SD)	8.51 (0.85)	8.55 (0.80)	8.53 (0.82)
FPG in mmol/L (SD)	9.60 (1.96)	9.58 (1.99)	9.59 (1.97)
2‐h postprandial blood glucose in mmol/L (SD)	14.8 (3.3)	14.7 (3.2)	14.7 (3.3)
eGFR in mL/min/1.73 m^2^ (SD)	132 (37.6)	133 (35.6)	132 (36.6)
SBP in mm Hg (SD)	128 (11.9)	127 (12.9)	128 (12.4)
DBP in mm Hg (SD)	80 (8.8)	79 (8.6)	80 (8.7)
Type of antidiabetic treatment*, *n* (%)			
Biguanides	203 (100)	203 (100)	406 (100)
Sulfonylurea	11 (5.4)	13 (6.4)	24 (5.9)
DPP‐4 inhibitors	5 (2.5)	6 (3.0)	11 (2.7)
Alpha‐glucosidase inhibitors	16 (7.9)	12 (5.9)	28 (6.9)
GLP‐1	9 (4.4)	7 (3.5)	16 (3.9)
Insulin	19 (9.4)	12 (5.9)	31 (7.6)
Duration of biguanides treatment in years (SD)	3.0 (4.1)	3.2 (3.9)	3.1 (4.0)

Abbreviations: BMI, body mass index; DBP, diastolic blood pressure; DPP‐4, Dipeptidyl‐peptidase 4; FPG, fasting plasma glucose; GLP‐1, glucagon‐like peptide‐1; HbA1c, hemoglobin A1c; PPG, postprandial glucose; SBP, systolic blood pressure.

* All subjects had stopped taking other antidiabetic treatments except biguanides before randomization.

Other baseline characteristics of the subjects, such as diabetes duration, diabetes complications, fasting plasma glucose, 2‐h PPG, HbA1c, estimated glomerular filtration rate, past antidiabetic treatment, and treatment duration were balanced between the two groups with no significant differences (Table 1).

### 3.2 Primary endpoint

For the main estimand analysis, the observed values after use of rescue medication or dosing cessation were first set as missing, and then multiple imputation was performed on all missing values. An MMRM analysis of covariance was used to evaluate the treatment difference analysis with the data after the multiple imputation stop. Based on the ITT analysis set, the least squares mean (LSM) changes in HbA1c from baseline to week 24 in the two groups were −1.08% in the bexagliflozin group and −1.10% in the dapagliflozin group, respectively. The difference between groups was 0.03% with 95% CI (−0.14% to 0.19%) and the upper limit was less than the prespecified margin of 0.4%, affirming the noninferiority criterion. Both the bexagliflozin group and the dapagliflozin group experienced clinically significant reductions in HbA1c levels. The results in the PP set were similar to those for the ITT set, with LSM changes in HbA1c from baseline to week 24 of −1.07% in the bexagliflozin group and −1.11% in the dapagliflozin group. The difference between bexagliflozin and dapagliflozin groups was 0.03%, with a two‐sided 95% CI of −0.14% to 0.21%.

The results of sensitivity analyses using the two supportive estimands (treatment policy and while on treatment strategy) also showed that the mean difference in HbA1c reduction between bexagliflozin group and dapagliflozin group was between 0.01% and 0.04%, with the upper limits of the 95% confidence intervals lower than the noninferiority margin of 0.4%, consistent with the results of the main estimand analysis.

**FIGURE 2:**
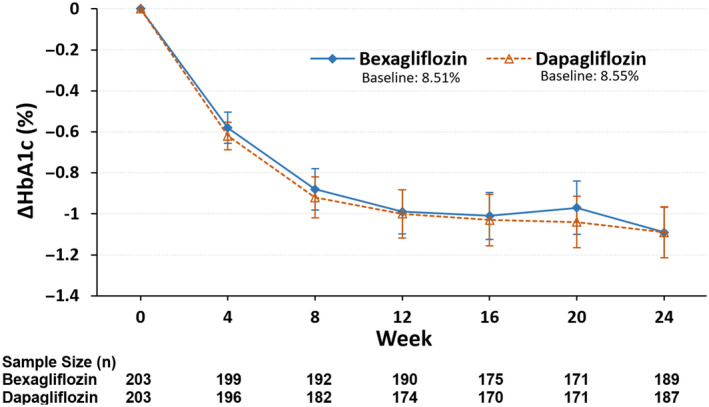
Change in glycated hemoglobin (HbA1c) from baseline as a function of time. Shown are means and 95% confidence intervals (CIs) for the individual arms and the difference.

The change in HbA1c as a function of time is shown in Figure 2.

### 3.3 Secondary endpoints

**FIGURE 3:**
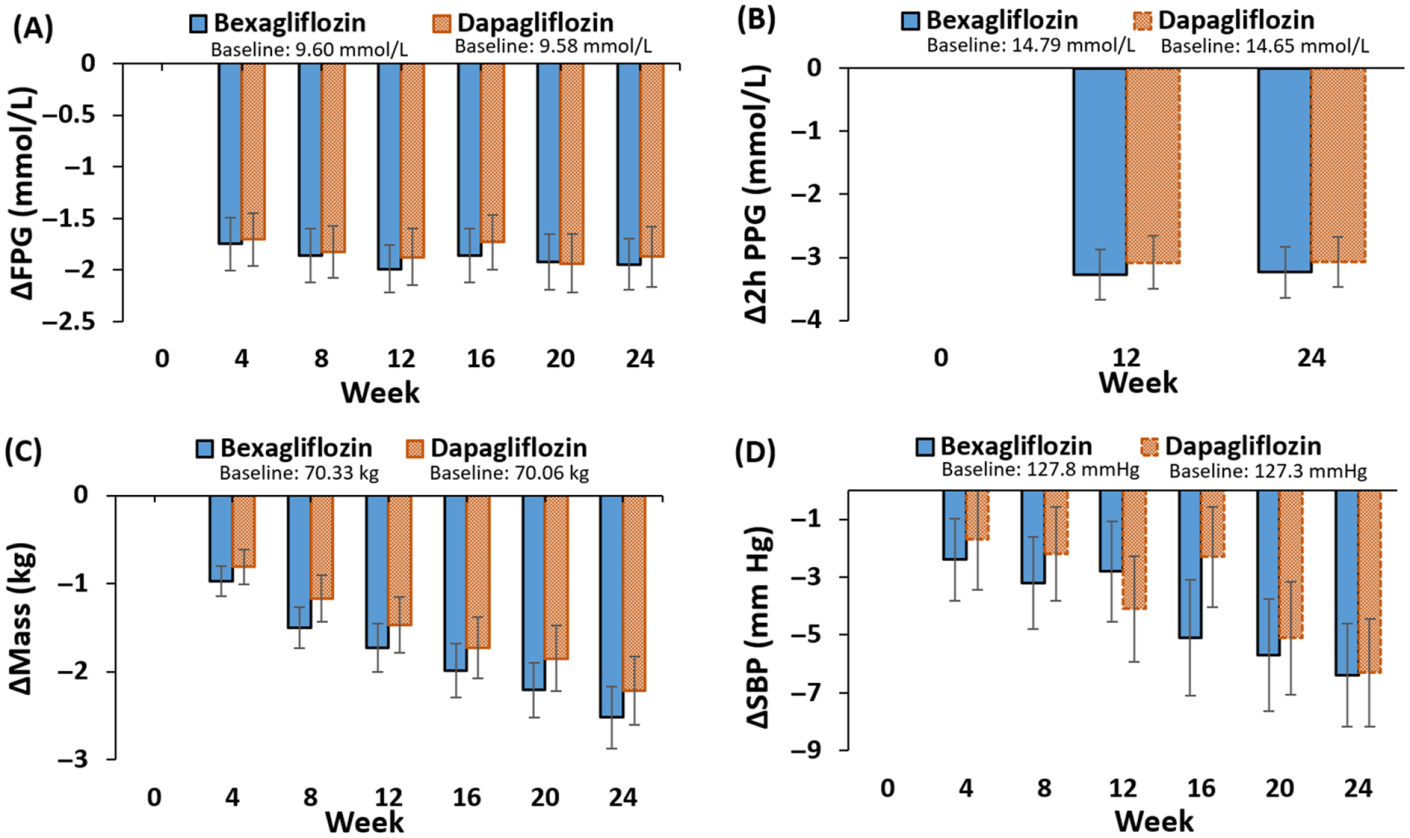
Change in (A) fasting plasma glucose (FPG), (B) 2‐h postprandial glucose (PPG), (C) body mass, and (D) systolic blood pressure (SBP) as a function of time. Shown are means ±95% confidence intervals (CIs).

Figure 3A and Table 2 show the mean change in fasting plasma glucose as a function of time. The mean change in FPG from baseline to week 24 was −1.95 mmol/L in the bexagliflozin group and −1.87 mmol/L in the dapagliflozin group, with an LSM difference of −0.12 mmol/L (95% CI −0.47 to 0.24; *p* = .5237) between the bexagliflozin group and the dapagliflozin group.

**TABLE 2:** Change from baseline in body mass, SBP, FPG, and PPG.

Variables	Bexagliflozin (*N* = 203)	Dapagliflozin (*N* = 203)
Body mass (kg)		
Week 24 *n*	189	186
Baseline body mass (SD)	70.33 (11.854)	70.06 (11.645)
Mean change from baseline (SD)	−2.52 (2.440)	−2.22 (2.702)
LSM Δ (SE)	−2.50 (0.189)	−2.19 (0.191)
Difference of LSM (SE)	−0.31 (0.268)	
95% CI for difference; *p*	(−0.842 to 0.214); 0.2432	
Systolic blood pressure (mm Hg)		
Week 24 *n*	189	187
Baseline SBP (SD)	127.8 (11.87)	127.3 (12.86)
Mean change from baseline (SD)	−6.4 (12.47)	−6.3 (13.00)
LSM Δ (SE)	−6.5 (0.91)	−6.3 (0.92)
Difference of LSM (SE)	−0.2 (1.30)	
95% CI for difference; *p*	(−2.71 to 2.39); 0.9031	
Fasting plasma glucose (mmol/L)		
Week 24 *n*	189	187
Baseline FPG (SD)	9.604 (1.9618)	9.582 (1.9924)
Mean change from baseline (SD)	−1.945 (1.7255)	−1.872 (2.0197)
LSM Δ (SE)	−1.918 (0.1281)	−1.802 (0.1291)
Difference of LSM (SE)	−0.116 (0.1819)	
95% CI for difference; *p*	(−0.4736 to 0.2415); 0.5237	
2‐h postprandial glucose (mmol/L)		
Week 24 *n*	192	187
Baseline PPG (SD)	14.786 (3.3313)	14.652 (3.1763)
Mean change from baseline (SD)	−3.238 (2.8540)	−3.069 (2.8061)
LSM Δ (SE)	−3.191 (0.1938)	−3.047 (0.1968)
Difference of LSM (SE)	−0.144 (0.2762)	
95% CI for difference; *p*	(−0.6873 to 0.3989); 0.6020	

Abbreviations: CI, confidence interval; FPG, fasting plasma glucose; LSM: least squares mean; PPG, 2 h post‐prandial glucose; SBP, systolic blood pressure.

For the change in 2‐h PPG concentration from baseline to week 24 (Figure 3B, Table 2), the mean changes were −3.24 mmol/L and −3.07 mmol/L in the bexagliflozin group and dapagliflozin group, respectively. The difference of the LSM between the bexagliflozin group and the dapagliflozin group was −0.15 mmol/L, with a 95% CI of −0.69 to 0.40 and a *p* value of .6020.

A decrease in mean body weight was observed in both groups (Figure 3C, Table 2). The average change in weight from baseline to week 24 was −2.52 kg for the bexagliflozin group and −2.22 kg for the dapagliflozin group. The difference in the LSM between the bexagliflozin group and the dapagliflozin group was −0.31 kg (95% CI −0.842 to 0.214); *p* = .2432.

Subjects in both arms exhibited a clinically meaningful decrease in SBP (Figure 3D, Table 2). The average change in SBP from baseline to week 24 was −6.4 mm Hg for the bexagliflozin group and −6.3 mm Hg for the dapagliflozin group, with a mean difference of −0.2 mm Hg (95% CI −2.71 to 2.39; *p* = .9031). Both arms also had a decrease in diastolic blood pressure, but the magnitude of the decrease was smaller than for SBP.

At week 24, 61 subjects (32.3%) in the bexagliflozin group and 59 subjects (31.6%) in the dapagliflozin group achieved HbA1c levels <7%, and 23 subjects (12.2%) in the bexagliflozin group and 21 subjects (11.2%) in the dapagliflozin group achieved HbA1c levels < 6.5%.

### 3.4 Safety

Treatment compliance was similar between the two cohorts, and the subjects tolerated the investigational products well. TEAEs occurred in 259 subjects (63.79%), including 127 subjects in the bexagliflozin group (62.56%) and 132 subjects in the dapagliflozin group (65.02%). The incidence of TEAEs did not differ significantly between the two arms. No deaths occurred during the trial. Sixteen subjects experienced at least one SAE, including nine subjects in the bexagliflozin group (4.43%) and 7 subjects in the dapagliflozin group (3.45%). AEs resulted in dosing cessation for two subjects in the bexagliflozin group (0.99%) and four subjects in the dapagliflozin group (1.97%). A total of six subjects (1.48%) withdrew from the study due to adverse events, including two subjects in the bexagliflozin group (0.99%) and four subjects in the dapagliflozin group (1.97%) (Table 3).

**TABLE 3:** Frequent or noteworthy adverse events.

Variables	Bexagliflozin *n* (%)	Dapagliflozin *n* (%)	Total *n* (%)
Number of subjects	203	203	406
Subject with at least one TEAE	127 (62.6)	132 (65.0)	259 (63.8)
Total TEAEs	322	325	647
AEs considered exposure related	69 (34.0)	61 (30.1)	130 (32.0)
Subjects with at least one SAE	9 (4.4)	7 (3.5)	16 (3.9)
SAEs considered exposure related	0	0	0
Subjects with AEs leading to dose cessation	2 (1.0)	4 (2.0)	6 (1.5)
Subjects with AEs leading to study withdrawal	2 (1.0)	4 (2.0)	6 (1.5)
Subjects with AE leading to death	0	0	0
Metabolism and nutrition disorders			
Hyperlipidemia	19 (9.4)	17 (8.4)	36 (8.9)
Hyperuricemia	12 (5.9)	11 (5.4)	23 (5.7)
Diabetic ketosis	10 (4.9)	10 (4.9)	20 (4.9)
Ketosis	8 (3.9)	9 (4.4)	17 (4.2)
Hypoglycemia	7 (3.5)	8 (3.9)	15 (3.7)
Hypertriglyceridemia	6 (3.0)	3 (1.5)	9 (2.2)
Dyslipidemia	2 (1.0)	6 (3.0)	8 (2.0)
Hypercalce,ia	3 (1.5)	4 (2.0)	7 (1.7)
Investigations			
Urine ketone body present	20 (9.9)	9 (4.4)	29 (7.1)
Blood parathyroid hormone increased	11 (5.4)	8 (3.9)	19 (4.7)
White blood cells urine positive	7 (3.5)	6 (3.0)	13 (3.2)
Blood uric acid increased	5 (2.5)	3 (1.5)	8 (2.0)
Urinary occult blood positive	5 (2.5)	2 (1.0)	7 (1.7)
Protein urine present	4 (2.0)	1 (0.5)	5 (1.2)
Infections and infestations			
Urinary tract infection	13 (6.4)	16 (7.9)	29 (7.1)
Upper respiratory tract infection	8 (3.9)	6 (3.0)	14 (3.5)
Gastrointestinal disorders			
Diarrhea	4 (2.0)	2 (1.0)	6 (1.5)
Cardiac disorders			
Myocardial ischaemia	1 (0.5)	4 (2.0)	5 (1.2)
Renal and urinary disorders			
Renal cyst	4 (2.0)	1 (0.5)	5 (1.2)
Blood and lymphatic system disorders			
Anemia	1 (0.5)	4 (2.0)	5 (1.2)

Abbreviations: AE, adverse event; SAE, serious adverse event; TEAE, treatment‐emergent adverse event.

The most commonly occurring (≥3%) TEAEs by preferred term were hyperlipidemia, hyperuricemia, diabetic ketosis, ketosis, and hypoglycemia in the system organ class (SOC) of metabolism and nutrition disorders; urine ketone body present, blood parathyroid hormone (PTH) increased, and white blood cells urine positive in the SOC of investigations; and urinary tract infection and upper respiratory tract infection in the SOC of infections and infestations. The incidence of urine ketone body present, based on positive urine ketone body results on urinalysis, was higher in the bexagliflozin group than in the dapagliflozin group, at 9.85% and 4.43%, respectively (Table 3). Except for the occurrence of urine ketone body present, the incidence of other TEAEs was closely similar between the bexagliflozin group and the dapagliflozin group (Table 3).

In terms of severity, most AEs were mild or moderate (Grade 1 or 2). Of the 16 subjects (3.9%) with TEAEs of Grade 3 or higher, 11 subjects were in the bexagliflozin group (5.42%) and five subjects in the dapagliflozin group (2.46%).

A total of 130 subjects had TEAEs deemed by investigators to be related to bexagliflozin or dapagliflozin treatment, with similar incidence rates in the bexagliflozin group (34.0%) and the dapagliflozin group (30.1%). More subjects in the dapagliflozin group (15.3%) than in the bexagliflozin group (11.3%) experienced an AESI. The AESI incidence rates (bexagliflozin vs. dapagliflozin) from high to low were urinary tract infection (6.40% vs. 8.37%), hypoglycemia (3.45% vs. 3.94%), major adverse cardiovascular events (1.48% vs. 0.99%), and falls and fractures (0.49% vs. 1.48%). All AESIs related to bexagliflozin or dapagliflozin treatment were Grade 1 or 2 according to the Common Terminology Criteria for Adverse Events scale, with no Grade 3 or higher events reported.

Four subjects ceased dosing investigational product due to SAEs, including one subject with a pulmonary tumor in the bexagliflozin arm, two subjects with cerebral infarctions, and one subject with stage IV ovarian cancer in the dapagliflozin arm. No treatment‐related SAEs were reported in either the bexagliflozin group or dapagliflozin group during the study.

Both groups showed a slight increase in red blood cell count (RBC), hematocrit, and hemoglobin compared to their respective baselines. Total cholesterol, low‐density lipoprotein cholesterol (LDL‐C), and high‐density lipoprotein cholesterol (HDL‐C) increased relative to baseline. Magnesium ion levels increased in both groups, with a greater increase in the bexagliflozin group (5.8%–8.7%) compared to the dapagliflozin group (2.7%–4.6%). Uric acid levels in both groups showed a downward trend with similar amplitude. The increase in PTH in the bexagliflozin group (5.42%) was higher than in the dapagliflozin group (3.94%). Both groups showed fluctuations in urea and blood urea nitrogen relative to baseline, with a slight increase in trend, and the number of observed values low. The changes in clinical laboratory parameters were either restored to their respective baselines or showed a trend toward recovery 2 weeks after discontinuing administration of investigational product (Table S2).

## 4 DISCUSSION

In this randomized, double‐blind, parallel, active‐controlled trial, both bexagliflozin and dapagliflozin produced significant and clinically meaningful reductions in HbA1c. For the primary endpoint of change in HbA1c from baseline to week 24, the main objective to demonstrate that bexagliflozin was noninferior to dapagliflozin was achieved. The similar reductions in HbA1c observed with bexagliflozin and dapagliflozin suggest that both SGLT2 inhibitors are effective in glycemic control. Moreover, the consistent outcomes between the ITT and PP sets reinforce the reliability of the study results, suggesting that the findings are likely to be applicable in broader clinical practice.

Secondary efficacy endpoint analyses showed results in both groups consistent with previous clinical trials, including a reduction in FPG and PPG, body weight, and SBP. East Asian populations are known to have higher postprandial glucose levels compared to non‐Asian populations.^20^, ^21^ Although a definitive explanation remains to be advanced, the difference has been attributed to potential factors including genetic predisposition, a more rapid decline in beta‐cell function, and dietary influences. Considering the increased prevalence of postprandial hyperglycemia in Asian patients with T2DM, monitoring and managing PPG is desirable for achieving optimal glycemic control. In this study, bexagliflozin demonstrated a significant reduction (3.24 mmol/L) in 2‐h postprandial glucose concentration.

In the renal proximal tubule, glucose reabsorption is linked to sodium reabsorption. By inhibiting SGLT2, sodium excretion and osmotic diuresis are promoted, leading to a decrease in plasma and extracellular fluid volume and a reduction in blood pressure. A clinically significant decrease in SBP was observed both in the bexagliflozin and dapagliflozin arms with an average change exceeding −6.00 mm Hg at week 24 from baseline. The results are consistent with published estimates of SGLT2 inhibitor effects from a meta‐analysis.^4^

The investigational products were generally well tolerated by the subjects in the bexagliflozin and dapagliflozin groups, with a similar overall incidence of AEs in both groups. The proportion of SAEs was low, with two subjects experiencing cerebral infarction in each group, and other SAEs occurring in only one subject in either arm. Four subjects (2%) in the dapagliflozin group withdrew from the trial due to AEs and two subjects (1%) in the bexagliflozin group. The most frequently occurring AEs of special interest were urinary tract infections (6.4% in the bexagliflozin arm; 8.4% in the dapagliflozin arm) and hypoglycemia (3.5% in the bexagliflozin arm; 3.9% in the dapagliflozin arm).

Urinary tract infections are a known class effect of SGLT2 inhibitors.^22^ The mechanism of action diverts blood glucose to urine, making the urine a rich source of nutrients and a favorable environment for bacteria, yeast and fungi growth. This can potentially lead to an increased risk of urinary tract infections (UTIs) in people with T2DM that take these medications. In this trial, 29 subjects experienced a UTI (7.1%) with 13 in the bexagliflozin group (6.4%) and 16 in the dapagliflozin group (7.9%). All UTIs that occurred in this trial were mild to moderate in severity, and there were no UTIs that were SAEs or led to dosing cessation. All recorded hypoglycemic events in this trial were mild and there were no serious events. The frequency of urine ketone body present, based on positive urine ketone body results on urinalysis, was higher in bexagliflozin arm compared to dapagliflozin arm, occurring in 9.85% and 4.43% of subjects, respectively.

The changes observed in laboratory parameters in this trial were previously reported consequences of exposure to SGLT2 inhibitors, consistent with previous clinical trial results. Both bexagliflozin and dapagliflozin decreased levels of AST and ALT. This is consistent with the results of large randomized controlled trials of similar drugs such as empagliflozin^23^ and canagliflozin.^24^ Treatment with SGLT2 inhibitors has been significantly associated with a decrease in ALT and AST levels when compared to placebo or active controls. This trial found an increase in total cholesterol, LDL‐C, and HDL‐C, which is consistent with the conclusions of meta‐analyses of SGLT2 inhibitors.^25^, ^26^ Net changes in lipid parameters were favorable (due to the improvement in ratio of HDL to LDL cholesterol) for cardiovascular outcomes in patients with diabetes. The elevations of serum magnesium and phosphate were consistent with literature reports,^27^ and the increase in phosphate concentration may be related to the increased reabsorption of phosphate by SGLT2 inhibitors.^28^ PTH also increased, and an increase in PTH concentration after taking SGLT2 inhibitors has been reported, possibly related to effects on the FGF23–1,25(OH) _2_D–PTH axis^28^, ^29^; PTH returned to baseline following dosing cessation. The decrease in serum uric acid is also an apparent class effect of SGLT2 inhibitors.^30^ The subjects in both groups experienced a slight increase in RBC, hematocrit, and hemoglobin compared to their respective baselines, which returned to baseline after discontinuation, as expected for the effect on RBCs and related parameters. A meta‐analysis of 40 randomized controlled trials found that SGLT2 inhibitors can increase hematocrit compared to placebo (weighted mean difference 2.67%, 95% CI 2.53% to 2.82%; *p* < .001),^31^ possibly due to the osmotic diuretic effect of SGLT2 inhibitors; another explanation is that erythropoietin production is increased.^32^ Increased hematocrit might be one of the underlying mechanisms of cardiovascular protection.^33^

Both treatments effectively and robustly improved blood glucose control in T2DM patients who failed metformin therapy, with virtually identical hypoglycemic effects observed for the two drugs. Both also had beneficial effects on weight loss, FPG, PPG, and SBP, which were favorable for disease control and patient health. At week 24, the change in HbA1c from baseline in the bexagliflozin group was not significantly different from that in the dapagliflozin group, with the upper limit of the 95% CI lower than the prespecified noninferiority threshold, achieving the main objective of the trial.

In terms of safety, drug‐related adverse events such as UTI and hypoglycemia found during the trial were similar to those recorded in previous SGLT2 trials, and no new drug‐specific adverse events were identified. The safety and tolerability of bexagliflozin and dapagliflozin were similar.

## FUNDING INFORMATION

The study was funded by Newsoara Biopharma Co., Ltd.

## CONFLICT OF INTEREST STATEMENT

Wei Zhang, Jinyu Chen, and Qian Zhu were employees of Newsoara Biopharma Co., Ltd. at the time of study conduct. The remaining authors declared that they do not have anything to disclose regarding funding or conflict of interest with respect to this manuscript.

## Supporting information


**Data S1.** Supporting Information.

## Data Availability

The authors confirm that the data supporting the findings of this study are available within the article [and/or] its Supplementary Materials.
