# Supplementary material for: Efficacy and safety of bexagliflozin compared with dapagliflozin as an adjunct to metformin in Chinese patients with type 2 diabetes mellitus: A 24‐week, randomized, double‐blind, active‐controlled, phase 3 trial
Source: J Diabetes. 2024 Apr 7;16(4):e13526. doi: 10.1111/1753-0407.13526 (PMC10999497; doi:10.1111/1753-0407.13526)
Supplement: Supplementary file 1 — Data S1. Supporting Information. [file JDB-16-e13526-s001.docx]

# Supplementary Figures and Tables

**Supplementary Table 1** Inclusion and Exclusion Criteria

| **Inclusion:** Each subject was required to meet the following criteria at the time of enrollment to be eligible for the study: |  |
| --- | --- |
| 1. To have been male or female adults with an age ≥ 18 years |  |
| 1. To have been negative on the urine pregnancy test and to have agreed to abstain from coitus or to use contraception throughout the study period until 30 days after the study drug was discontinued if a female of childbearing potential |  |
| 1. To have had a diagnosis of T2DM with HbA1c values of 7.5 – 11% (inclusive) at the screening and have had HbA1c values of 7.0 – 10.5% (inclusive) at the enrollment |  |
| 1. To have been treated with a stable dose of ≥ 1500 mg/day metformin as a monotherapy along with diet and exercise counseling for at least 8 weeks at the time of screening |  |
| 1. To have had a BMI between 19 and 35 (inclusive) kg/m^2^ at the time of screening |  |
| 1. If applicable, to have been receiving stable doses of treatment for lipid abnormalities and/or hypertension for 30 days before the screening. Subjects not taking any medications for lipid abnormalities and/or hypertension within 30 days before the screening or those who displayed lipid and/or blood pressure abnormalities during the screening period but were expected to remain on a stable therapeutic regimen during the study, as determined by the investigators, were also eligible to participate in the study. |  |
| 1. To have been willing and able to return for all clinic visits and to complete all study required procedures, including SMBG measurements. |  |
| 1. To have complied with the drug administration requirements for the study drug, with drug consumption during the run-in period being between 80% and 120% of expected (inclusive). |  |
| **Exclusion:** Potential subjects who exhibited any of the following characteristics were to be excluded from the study: | |
| 1. A history of diabetes insipidus | |
| 1. A diagnosis of type 1 diabetes, or maturity-onset diabetes of the young (MODY) in adolescents or young adults, or secondary diabetes | |
| 1. A history of poorly controlled diabetes symptoms that would prevent participation in this trial, including but not limited to significant polyuria and polydipsia in the 3 months before screening, and weight loss exceeding 10% | |
| 1. A history of genitourinary infections requiring treatment ≥ 3 times in the 6 months before screening, or of genitourinary infection within 6 weeks before screening | |
| 1. Two or more consecutive fasting self-monitoring of blood glucose (SMBG) values ≥ 250 mg/dL (13.9 mmol/L) with clinical signs or symptoms of hyperglycemia, including weight loss, blurred vision, thirst, increased urination, or fatigue, before randomization (during the run-in period) | |
| 1. A history of diabetic ketoacidosis or hyperosmolar non-ketotic coma within 6 months before screening | |
| 1. A history of severe osteoporotic fracture | |
| 1. Poorly controlled hypertension: systolic blood pressure ≥ 160 mmHg and/or diastolic blood pressure ≥ 100 mmHg | |
| 1. A history of surgery causing unstable weight, or planned surgery during the study | |
| 1. Any unstable endocrine, psychiatric, or rheumatic disease as judged by the investigator | |
| 1. According to the investigator's judgment, a history suggesting the subject may be at risk of dehydration or fluid depletion, which may affect the interpretation of efficacy or safety data | |
| 1. A history of alcohol or drug abuse within the past 6 months. | |
| 1. A history, within 6 months prior to screening, of: | |
| - Myocardial infarction | |
| - Cardiac surgery or vascular reconstruction (coronary artery bypass grafting/percutaneous coronary intervention) | |
| - Unstable angina | |
| - Unstable congestive heart failure | |
| - New York Heart Association congestive heart failure III or IV | |
| - Transient ischemic attack or severe cerebrovascular disease | |
| - Unstable or poorly controlled arrhythmias | |
| 1. Unstable or rapidly progressing kidney disease | |
| 1. Congenital renal glycosuria | |
| 1. A history of major liver disease, including but not limited to chronic active hepatitis and/or significant liver function abnormalities, subjects with ALT and/or AST levels greater than or equal to 3 times the upper limit of normal and/or total bilirubin levels greater than or equal to 2 times the upper limit of normal; or subjects with a history of severe liver or biliary disease or drug-induced liver toxicity | |
| 1. A history of active hepatitis B or C, except in the following cases: those with positive HBsAg and HBV DNA titers not exceeding the upper limit of normal at the study center can be included; those with positive HCV antibodies and HCV RNA titers not exceeding the upper limit of normal at the study center can be included | |
| 1. A history of hemoglobinopathy, except for sickle cell trait or mild thalassemia, or chronic recurrent hemolysis | |
| 1. A history of uncontrolled thyroid dysfunction, defined as thyroid-stimulating hormone (TSH), free triiodothyronine (FT3), or free thyroxine (FT4) levels outside the normal range; or if TSH is outside the normal range but FT3 and FT4 are within the normal range, the subject can be included if the investigator determines that there is no clinical significance | |
| 1. Any laboratory test result meeting the following criteria: | |
| - Male hemoglobin ≤ 10 g/dL (≤ 100 g/L); female hemoglobin ≤ 9.0 g/dL (≤ 90 g/L) | |
| - Parathyroid hormone (PTH) > 1.5 times the upper limit of normal | |
| - Creatine kinase ≥ 3 times the upper limit of normal | |
| - Serum calcium < 1.9 mmol/L or > 2.75 mmol/L (< 7.6 mg/dL or > 11.0 mg/dL), or if only ionized calcium can be detected at the study center, it should be excluded if it exceeds the normal range at the study center | |
| - Serum phosphate < 0.65 mmol/L or > 1.8 mmol/L (< 2.0 mg/dL or > 5.6 mg/dL) Fasting triglycerides > 5.64 mmol/L (500 mg/dL) | |
| - eGFR < 60 mL/min/1.73m^2^ (calculated using the modified Chinese simplified MDRD formula: 175 × [(serum creatinine (μmol/L)/88.4)]^-1.234^ × [age (years)]^-0.179^ × 0.79 (female) or ×1 (male)); or male subjects with serum creatinine levels ≥ 1.50 mg/dL (133 μmol/L) or female subjects with serum creatinine levels ≥ 1.40 mg/dL (124 μmol/L). | |
| 1. A history of blood transfusion, blood product treatment, or loss of more than 400 milliliters of blood (including blood donations) within 3 months prior to screening visit. | |
| 1. A history of malignant tumors within 5 years prior to screening visit (excluding treated basal cell carcinoma or squamous cell carcinoma). | |
| 1. A history of organ transplantation, positive test for HIV, or other immune deficiencies. | |
| 1. A history of intolerance, contraindication, possible allergy or hypersensitivity reaction to the ingredients of bexagliflozin tablets, dapagliflozin tablets, metformin tablets, or similar products of these three drugs. | |
| 1. A history of any antidiabetic treatment (except for orally administered hypoglycemic drugs allowed by the protocol), including traditional Chinese medicine for more than 14 days (continuous or intermittent) within 12 weeks prior to screening visit. | |
| - Additionally, a history of any antidiabetic treatment, including traditional Chinese medicine (except for orally administered hypoglycemic drugs allowed by the protocol), received at any dose within 8 weeks prior to screening visit. | |
| 1. A history of treatment with SGLT-2 inhibitors or participation in SGLT2 inhibitor clinical trials within 12 weeks prior to screening visit. | |
| 1. A history of alternative or chronic systemic corticosteroid therapy, defined as the use of any dose of systemic corticosteroids for more than 4 weeks within 3 months prior to screening visit. | |
| 1. A history of weight loss drug consumption within 30 days prior to screening visit, including but not limited to sibutramine, phentermine, orlistat, rimonabant, levomethamphetamine, methylpropionic acid, methamphetamine, and / or phendimetrazine . | |
| 1. A history of long-term consumption of Chinese medicine with nephrotoxic effects (such as aristolochic acid) within 30 days of screening. | |
| 1. Pregnancy or lactation in women, or an unwillingness to use contraception by men or women of childbearing potential for the period of the study and for 30 days following the last administration of the study drug. | |
| 1. A history of exposure to any other investigational drug or participation in any interventional clinical trial within 30 days prior to screening. | |
| 1. Any other factors or clinical abnormalities judged by the investigator that could have affected the efficacy or safety evaluation of this study. | |
| Before entering the randomization visit, all non-laboratory-related inclusion and exclusion criteria were to continue to be met until the randomization visit. At the randomization visit, the inclusion and exclusion criteria were to be evaluated again, and the eligible subjects were to be randomly assigned to either treatment group. | |

Abbreviations: ALT, alanine aminotransferase; AST, aspartate aminotransferase; FT3, free triiodothyronine; FT4, free thyroxine; HBsAg, Hepatitis B surface antigen; HBV DNA, Hepatitis B virus deoxyribonucleic acid; HCV, Hepatitis C virus; HCV RNA, Hepatitis C virus ribonucleic acid; HIV, human immunodeficiency virus; MDRD, modification of diet in renal disease; MODY, maturity-onset diabetes of the young; PTH, parathyroid hormone; SMBG, self-monitored blood glucose; TSH, thyroid stimulating hormone.

**Supplementary Table 2** Laboratory parameters

| **Parameter** | **Bexagliflozin** | **Dapagliflozin** |
| --- | --- | --- |
| **Erythrocytes [× 10^12^ /L] (SD)** | 4.87 (0.45) | 4.78 (0.47) |
| **Erythrocytes Δ^*^** | W24:↑3.1%; W26:↑0.6% | W24:↑2.5%; W26:↓0.2% |
| **Hematocrit [%] (SD)** | 43.4 (3.6) | 42.6 (4.1) |
| **Hematocrit Δ** | W24:↑4.3%; W26:↑1.2% | W24:↑3.7%; W26:↑0.2% |
| **Hemoglobin [g /L] (SD)** | 147 (14) | 144 (15) |
| **Hemoglobin Δ** | W24:↑2.4%; W26:↓0.1% | W24:↑1.9%; W26:↓1.0% |
| **ALT [U/L] (SD)** | 24.3 (16.1) | 22.9 (12.1) |
| **ALT Δ** | W24:↓13.6%; W26:↓0.2% | W24:↓14.8%; W26:↓12.0% |
| **AST [U/L] (SD)** | 21.2 (8.6) | 20.5 (6.3) |
| **AST Δ** | W24:↓8.0%; W26:↓1.9% | W24:↓8.3%; W26:↓9.1% |
| **AKP [U/L] (SD)** | 82.1 (23.9) | 84.4 (25.7) |
| **AKP Δ** | W24:↓9.3%; W26:↓7.8% | W24:↓11.4%; W26:↓10.5% |
| **Magnesium [mmol/L] (SD)** | 0.83 (0.16) | 0.85 (0.14) |
| **Magnesium Δ** | W24:↑9.2%; W26:↑1.3% | W24:↑5.1%; W26:↓0.4% |
| **Phosphate [mmol/L] (SD)** | 1.13 (0.16) | 1.14 (0.19) |
| **Phosphate Δ** | W24:↑6.2%; W26:↑2.9% | W24:↑3.5%; W26:↑0.6% |
| **Bicarbonate [mmol/L] (SD)** | 25.2 (2.8) | 25.3 (2.9) |
| **Bicarbonate Δ** | W24:↓6.9%; W26:↓2.6% | W24:↓5.5%; W26:↓4.6% |
| **Carbon Dioxide [mmol/L] (SD)** | 25.9 (3.2) | 26.2 (5.8) |
| **Carbon Dioxide Δ** | W24:↓2.7%; W26:↑3.4% | W24:↓1.3%; W26:↓0.7% |
| **Uric Acid [**µ**mol/L] (SD)** | 309 (78.3) | 307 (81.9) |
| **Uric Acid Δ** | W24:↓3.7%; W26:↓2.3% | W24:↓3.7%; W26:↑4.3% |
| **PTH [pmol/L] (SD)** | 4.73 (2.10) | 4.56 (2.06) |
| **PTH Δ** | W24:↑7.0%; W26:↓3.0% | W24:↑1.8%; W26:↓10.3% |
| **TC [mmol/L] (SD)** | 4.81 (0.93) | 4.68 (1.03) |
| **TC Δ** | W24:↑2.3%; W26:↑1.7% | W24:↑2.6%; W26:↑3.8% |
| **LDL-C [mmol/L] (SD)** | 2.86 (0.81) | 2.75 (0.87) |
| **LDL-C Δ** | W24:↓0.4%; W26:↓2.1% | W24:↑0.9%; W26:↑1.0% |
| **HDL-C [mmol/L] (SD)** | 1.21 (0.29) | 1.23 (0.28) |
| **HDL-C Δ** | W24:↑5.4%; W26:↑4.0% | W24:↑5.0%; W26:↑5.3% |
| **TG [mmol/L] (SD)** | 1.84 (1.06) | 1.88 (1.01) |
| **TG Δ** | W24:↓5.3%; W26:↑1.4% | W24:↓4.8%; W26:↑0.1% |
| **eGFR [ml/min/1.73m] (SD)** | 132 (37.3) | 133 (35.3) |
| **eGFR Δ** | W24:↓3.3%; W26:↓1.3% | W24:↑2.2%; W26:↑3.7% |
| **Creatinine [**µ**mol/L] (SD)** | 61.5 (15.7) | 59.7 (15.2) |
| **Creatinine Δ** | W24:↑1.8%; W26:↓0.9% | W24:↓2.0%; W26:↓2.8% |
| **Urea [mmol/L] (SD)** | 5.39 (1.23) | 5.24 (1.31) |
| **Urea Δ** | W24:↑15.2%; W26:↑7.1% | W24:↑8.6%; W26:↑6.9% |
| **BUN [mmol/L] (SD)** | 5.14 (1.43) | 5.71 (1.82) |
| **BUN Δ** | W24:↑17.5%; W26:↑4.5% | W24:↑13.3%; W26:↑15.4% |

* Δ stands for change from baseline to week 24 (W24) or week 26 (W26) in percent.

Abbreviations: ALT, alanine aminotransferase; AKP, alkaline phosphatase; AST, aspartate aminotransferase; BUN, blood urea nitrogen; eGFR, estimating glomerular filtration rate; HDL-C, high density lipoprotein cholesterol; LDL-C, low density lipoprotein cholesterol; PTH, parathyroid hormone; TC, total cholesterol; TG, triglycerides.
